# Medical Society of the State of New York

**Published:** 1875-02

**Authors:** 


### MISCELLANEOUS.

### Medical Society of the State of New York.

### SIXTY NINTH ANNUAL SESSION.

*First Day—Morning Session.*—The Society met, pursuant to statute, at Albany, on Tuesday, February 2d, 1875, at 11 o'clock A. M., in the Common Council Chamber.

The meeting was called to order by the President, Dr. George J. Fisher, of Sing Sing, and a prayer was offered by the Rev. Doctor Bartlet.

The President then delivered his inaugural address, embracing a variety of topics of interest to the Society and the profession throughout the State.

At its conclusion Dr. A. Jacobi moved the appointment of a committee to consider the suggestions of the president, with instructions to report on the same at as early an hour as possibile, especially that portion relating to changing the time and place of meeting.

The following committees were then appointed by the President:

*On the President's Address.*—Dr. A. Jacobi, Bates and Jenkins.

*On Credentials.*—Drs. W. C. Wey, Thomas F. Rochester and Thompson Burton.

*On Arrangements and Receptions.*—Drs. F. Hyde, Wm. H. Bailey, and N. C. Husted.

*Business Committee.*—J. V. P. Quackenbush, D. B. St. John Roosa, and Ellsworth Eliot.

*On Ethics.*—Drs. Thos. Hun, E. Krackowizer, and J. G. Adams.

Dr. D. B. St. John Roosa, of New York, next read a paper on "Hysterical Amblyopia, with Concentric Limitation of the Visual Field." A discussion followed, in which Drs. A. Jacobi, Squibb, Matthewson, and Pooley joined; the former calling attention to the distinction to be made between hysterical and simulated diseases.

A communication was read from Governor Tilden, inviting the Society, its guests and the members of the Albany Co. Med. Soc., to the executive mansion on the following evening. On motion, the invitation was accepted.

Several papers, including a biographical sketch of the late Prof. James McNaughton, of Albany, were read by title and referred to the Committee on Publication.

Dr. Hyde read a paper on strangulated hernia, and was followed in discussion by Dr. Squibb, who called attention to intoxication by conium as a means of producing relaxation of the muscular system to a degree which favored taxis.

Dr. Govan moved the appointment of a committee to invite the Governor, State officers, and members of the Legislature to be present during the sessions of the Society. Carried.

Dr. Paddock, of the Massachusetts State Medical Society; Drs. Morgan, Newton, and Rogers, of Vermont, and Dr. Hoar, of Maine, were presented, after which the Society adjourned to 3.30. P. M.

---

*Afterroon Session.*—The Society having assembled pursuant to adjournment, the President announced the following

COMMITTEES.

Committee on Nomination of Officers for the ensuing years, 1st District, Dr. Thomas Addis Emmet; 2d, Dr. J. Foster Jenkins; 3d, S. Oakley Vanderpoel; 4th, Dr. Thompson Burton; 5th, Dr. Alonzo Churchill; 6th, Dr. Wm. C. Wey; 7th, Dr. F. Hyde; 8th, Dr. E. M. Moore.

Committee to invite the officers of the State government to be present at the meetings; Drs. William Govan, Thompson Burton, and Arthur Wolfe.

Dr. T. R. Pooley, of New York, read a paper entitled "Foreign Bodies in the Eye." He detailed several cases illustrating the modes of production and effects of such injuries, together with the method of operating. Enucleation of the injured eye was insisted on as the only safe way of preventing sympathetic inflammation of the sound eye when we were perfectly sure of the presence of a foreign body of any sort which we are not able to remove.

Dr. Vedder, of Schenectady, related a case, the results of which he thought opposed Dr. Pooley's opinion of the necessity for enucleation.

Dr. Agnew, of New York, quite agreed with the writer of the paper on the necessity for enucleation under the circumstances he mentioned. He believed that the form of sympathetic disease induced in a fellow eye, even when the trouble is not in the ciliary region, is so insidious and intractable that the only way to prevent loss of sight in the uninjured eye is to remove the diseased organ.

Dr. Roosa, of New York, did not consider the absorption of the crystalline, as mentioned by Dr. Vedder, a sufficient cure, nor could he consider it justifiable to leave the injured eye with its foreign contents *in situ.*

Dr. Vedder said the case came under his observation twenty years ago, and had as yet had no consecutive trouble.

Dr. Chapman, of Medina, mentioned a case reported in *The Medical Record* of May 1, 1873, by Dr. Knapp, in which a diseased ball had been removed. The patient had been seen by him the day before, and had suffered no return of the affection.

Dr. Charles H. Porter, Treasurer, submitted his report, which was referred to an auditing committee composed of Drs. Ferguson, Mynders and Lewis.

Dr. J. V. P. Quackenbush, of Albany, read the histories of two singular cases of what he called "subperitoneal cystic tumor."

In the first a tumor presented posteriorly to the cervix uteri in a woman in labor, and was gradually forced downwards in front of the child's head. When punctured it yielded a considerable quantity of serum, and relieved the labor of an obstacle to its completon. The second case was a young woman, who had, not long before the doctor saw her, arrived in this country, with a history and symptoms that strongly indicated pregnancy. A careful examination proved the fallacy of this supposition as well as that of ovarian tumor. The patient died, and a cyst was found between the abdominal walls and the peritoneum lining it, which contained a large quantity of clear fluid. The uterus and its appendages were normal and healthy.

Dr. A. Jacobi, of New York, commenting on the novelty of these cases, said that there appeared to him to be but two or three ways of explaining them; one was the occurrence of hemorrhage, the hemorrhagic mass being encysted, the clot absorbed, and only a serous fluid remaining, but in such case we would have the coloring matter of the blood remaining. Another cause would be the closure of a gland duct and accumulation of the secretion in quantity to form a cyst, but he was unaware of any glandular organs in these localities. The third and most probable cause was connected with errors in fœtal developement; of these some anomalous development of the cords of Muller, he thought, might be the most probable cause, but declined to give any decided opinion.

Dr. Thomas Addis Emmet, of New York, read a very practical paper on the "Treatment and Removal of Fibroids from the Uterus by Traction." The interesting point in the paper was the mode of formation of a pedicle in these cases.

Dr. Benedict, of Onondaga, discussed the paper.

Dr. S. O. Vanderpoel, of New York, read a portion of a paper on "Disinfection and disinfectants," and was followed by

Dr. E. Squibb, of Brooklyn, who presented to the Society a very valuable paper on "Salicylic Acid," a new product of the action of the carbonic acid on phenol, which is just commencing to excite a great deal of attention as a disinfectant and anti-ferment.

Dr. Castle, of New York, mentioned the price for which the acid, made by Kolbé's process, is being sold in Dresden.

The following papers were read by title, and on motion, referred to the Committe on Publication:—

"Fracture of the Skull with Depression, followed by Hemiplegia; Result of Trephining in Remedying the Paralysis." By Dr. R. W. Pease, of Syracuse.

Report of Dr. H. S. Chubbuck, Delegate to the American Medical Association.

Report of Dr. Hiram Corliss, of Greenwich, Washington Co., Delegate to the Maine and Massachusetts State Medical Societies. Read in full, together with a communication from the "Veteran member," regretting that ill-health prevented his attendance.

On motion of Dr. Vanderpoel, the president was requested to telegraph the sympathy of this Society to Dr. Corliss.

Dr. Ed. H. Parker offered a petition of an aggrieved member. It was referred to the Committee on Ethics.

On motion, it was decided to meet Wednesday morning, at half-past nine o'clock.

Recess until half-past seven.

*Evening Session.*—The Society met as agreed, at half-past seven, to listen to a paper by

Dr. John P. Gray, of Utica, on "Pathological Changes that Occur in Nervous Diseases, especially Insanity." The most notable feature were the use of the term "fatty involution" to express a variety of degenerative changes, and the illustration of the pathological conditions mentioned in the paper, by means of microphotographs projected by means of a calcium light upon a screen. After interesting the audience for an hour and a half, he was followed by a lecture by Dr. Louis Ellsberg, of New York, on Acoustics.

Many interesting experiments were made to illustrate the production of sound, its character as regards pitch, *timbre*, the mechanism of the human voice, etc. Both gentlemen received a unanimous vote of thanks, and the Society adjourned at ten o'clock, to meet on Wednesday morning at the hour appointed.

---

*Second Day—Morning Session.*—The Society met pursuant to agreement, at half-past nine o'clock, and the meeting was opened with prayer by the Rev. Anson J. Upson, of Albany.

### MISCELLANEOUS.

The Committee on Reception and arrangements reported that additional members had arrived and were present by invitation. The report was received and the gentlemen introduced to the Society.

The minutes of Tuesday's session were then read and approved.

Dr. E Squibb, senior censor for the Southern district, reported they had examined for license, Dr. Ezra S. McClellan, and recommended him for a diploma, and that he be now declared a licentiate of the Society. The report was adopted.

Prof. Dalton, of the New York County Medical Society, then addressed the Society on the subject of experimentation on animals for physiological and scientific purposes. He said that eight years ago Mr. Henry Bergh, of New York, commenced a series of newspaper articles, an onslaught on the practice, and it was subsequently followed up by the introduction of a bill in the Legislature for the organization of the Society for the Prevention of Cruelty to Animals. The Medical Society, in 1867, had memorialized the Legislature and procured a modification of this bill, so that experimentation on animals in the interests of science had been continued. Last summer, however, Mr. Bergh had renewed his attacks on the practice, as before, in a series of newspaper articles, published in the *New York Evening Post,* and his evident object was to place the Society under the control of himself and agents. The speaker then referred to the importance of the practice in determining the nature of various diseases, and the favorable results that have been realized, and urged that the Society take some action reaffirming the action of the Medical Society in 1867. With this object in view he offered a series of resolutions.

A discussion followed, in which Drs. B. F. Sherman and Squibb participated, and the preamble and resolutions were adopted unanimously.

Dr. J. G. Adams stated subsequently that he had held some conversation with Mr. Beach, the attorney of Mr. Bergh, and was informed that the latter had no intention of making an application to the Legislature for a change of the law; he was content to carry on the war with Dr. Dalton in the newspapers.

Dr. Vanderpoel believed that this was only a change of *venue;* that finding the profession ready and able to oppose him successfully, he was intending to interest public opinion in the matter so that the law may eventually be repealed.

Drs. J. F. Kendall, Moreau Morris, and B. F. Sherman were named a committee to invite the Health Committee of the Senate and Assembly to be present at the afternoon session of the Society, to hear the report of Dr. Bell on Drainage in this State.

Dr. Thomas F. Rochester, from the Committee on Prize Essays, reported but two competitive papers had been received for the McCosh prize on the designated subject for 1875. viz.: "School hygiene in reference to the Physiological Relations of Age and Sex to Mental and Physical Education." One of these had the motto, "The quality of the brain is the key to human knowledge," and was a good, practical, common-sense article, well worthy of publication. The other bore the motto, "Salut populi—suprema lex," a scholarly and philosophical essay, for which the prize was awarded to Alexander Hutchins, A. M., M. D., of No. 796 De Kalb Avenue, Brooklyn. No essays were received for the Brinsmade prize.

Dr. Mary Putnam-Jacobi delegate for the Medical Society of the County of New York, read a paper entitled, "Effects of the Nitrate of Silver on Epithelial and Gland Cells," accompanied with microscopic preparations. The paper embraced references to the literature of the subject, and detailed the results of numerous experiments on animals and freshly removed portions of human tissues.

Dr. Henry D. Noyes, in discussing the paper, referred to the assistance rendered by the nitrate of silver in the study of the structure of the cornea.

Dr. J. Marion Sims, of New York, made some very interesting remarks on the subject of "Utero-Gastrotomy;" saying, that having had the honor of reading a paper at the last annual meeting on the removal of intra-uterine fibroids by enucleation, he now proposes to speak of the removal of larger uterine fibroids by abdominal section, whether intra-uterine, interstitial, or extra-uterine in character. This operation is now on its trial. It stands where ovariotomy did twenty years ago. It has the same opposition to encounter, and will doubtless achieve the same victory. In this country in has been performed successfully by Kimball, Burnham, Boyd, Storer, Darby; in England by Charles Clay, Fletcher, and very recently by Lawson Tait. Koeberle, of Strasbourg, has cured four out of six cases, while Péan, of Paris, gives us the minute histories of eleven cases, with seven cures, and since the publication of his work, his pupil Urdi has published a work in which he says, that the whole number of Péan's operations up to the present time is twenty, with fifteen cures.

Dr. Sims has recently operated twice for the removal of the uterus, with large fibroid, by abdominal section. The first case was in a feeble state from excessive loss of blood. During the separation of a large fold of intestine from the surface of the tumor, the capsule of the tumor was torn up, large venous sinuses were opened, and the patient suddenly lost about sixteen ounces of blood. She never rallied, and died from the shock and loss of blood in thirty-five or forty minutes after the operation.

The second case had lost large quantities of blood and was quite anæmic, but was thought to be a favorable case for operation. It was done on the 19th of November, according to Péan's method. The patient died in seventy-six hours, of septicæmia. Examination, post-mortem, showed the pedicle in a sloughing condition below the wire clamp; the slough extending along the line of incision in the abdominal parietes, and on top of the bladder, and in the broad ligaments. There were eighteen ounces of bloody serum in the peritoneal cavity. Péan's method of operating is to make a pedicle of the supra-vaginal portion of the cervix, and to draw this out through the lower edge of the abdominal section by clamp, as in ovariotomy. He transfixes the crevix by a double wire, ties one on each side of the crevix, inclosing the broad ligament on its respective side in the wire. Dr. Sims employed Péan's method in both his cases, but would not use it again; but he advocates the use of the actual cautery. He exhibited a clamp écraseur on the principle of Nott's [and Isaac E. Taylor's], by which he would compress the broad ligament on one side near the body of the uterus, and then sever the ligament with the cautery down to its junction with the crevix. The same method is to be followed on the side, and it only remains to cut the tumor from the supra-vaginal cervix and cauterize the surface. The several cauterized portions are then dropped into the peritoneal cavity, when, in spite of the eschar, they unite at once by adhesive inflammation to the surfaces with which they lie in contact.

Dr. Sims then exhibited an automatic alcohol blow-pipe for heating the cautery irons.

Dr. E. M. Moore, of Rochester, said his views with regard to operations which required the opening of the abdominal cavity had, for several years, been undergoing considerable change, and there seems to be some truth in the idea that it may be as safe or safer to perform gastrotomy for uterine than for ovarian tumors, owing to the adhesions which are so likely to exist in the latter case.

While it is no trifling matter, or an operation to be done without good and urgent occasion, surgeons have to a great extent got over the fear of opening this cavity. A large number of unfavorable results are undoubtedly from septicæmia. Since Kimball, of Lowell, performed the first operation of this character, the proceedure has probably been resorted to more often than the profession were aware. In the case of Dr. Kimball the patient recovered. Last summer Dr. Moore said he had a case of uterine fibroid on which he operated successfully, in which the tumor weighed seventeen pounds. The operation was a modification of the one introduced by Prof. Miner, of Buffalo, in cases of ovariotomy, and called by him ovariotomy by enucleation.* In this case a pedicle was created by separating a portion of the serious membrane from the surface of the uterus and tumor and bringing it into the abdominal wound, where it was retained, as in the ovariotomy, and formed a cup which received the blood which might escape, and the discharges, and thus prevented their entrance into the abdominal cavity.

Prof. E. R. Peaslee, of New York, called attention to some points in Dr. Sims remarks. His own experience with this operation commenced one week after Dr. Kimball made his operation. The success or failure of the operation would depend upon a variety of causes, and was at best, even in the opinion of Dr. Sims, a dangerous one. Péan does not operate indiscriminately, but he has operated in a case of fibrocystic tumor even when the woman was nearly exhausted, and in some cases of fibroma with ascites, even

---

* See Trans. of the Society for 1872, page 187, and *Buffalo Med. and Surg. Journal* of June, 1869.

when the patient was nearly moribund. Dr. Peaslee has seen but two cases in which he thought the operation was advisable, but did not wish to be understood as opposing it. He was perfectly willing to undertake it when the indications were fulfilled.

Dr. Hurd, of Brooklyn, wished to ask Dr. Sims if he was aware of any objection to the use of the galvano-cautery in the separation of the mass.

Dr. Sims declared his readiness to use it, but, at the same time, would not neglect to put a clamp on the broad ligament to prevent hemorrhage.

Dr. Walter Kempser, delegate from Wisconsin, and formerly attached to the Utica Asylum, was introduced and made some remarks.

Dr. C. R. Agnew, of New York, read a paper on "Certain Diseases of the Eye," and their treatment by a modified operation by canthoplasty.

Dr. Geo. T. Stevens, of Albany, reported a "Case of Sarcoma of the Ciliary Body."

Dr. L. D. Bulkley, of New York, an invited guest, read a paper on the "Relation of the Urine to the Diseases of the Skin."

Dr. A. Jacobi, chairman of the Committee on the President's Address, presented the report.

The Committee depreciated the disposition on the part of specialists to publish so many journals devoted solely to their branches. This could not but have the effect of diverting from well established journals, devoted to general medicine and surgery, matters which ought to claim the attention of general practitioners. A few special journal were all that were required, and these might be of the nature of reviews, in which one could find the summary of current literature on special subjects, such as therapeutics, etc.

Regarding the tendency on the part of young men to practice specialties, the committee were equally decided. The tendency was to degrade the general practitioner in the estimation of the public. It was too often forgotten that most of our men who have become eminent in certain branches, were doctors before they became specialists.

The proposition of the President respecting the appropriation of not more than fifty copies of the Transactions for circulation among leading medical journals at home and abroad was approved; as was likewise the suggestion regarding memorial leaves to be incorporated in the Transactions; and suitable resolutions to that effect were, subsequent to the reading of the report, adopted by the Society.

The necessity for a codification of the laws relating to the profession was at the present stage of the existence of the Society, of very great importance, and the committee recommended that the matter be placed in the hands of a competent lawyer. Observations were made on the inefficiency of the law passed by the

Legislature at its last session, and the suggestion of the President that an effort should be made to establish a law like the one in Great Britain, endorsed.

The points of chief interest in the report of the committee related to the time and place of meeting, and the election of permanent members. Respecting the first, the committee recommended that the time of meeting be changed to the third Tuesday in June, and that a committee be appointed by the Chair to take measures, if necessary, to insure the acquiescence of the Legislature in the change.

After an animated discussion, a resolution to that effect was carried.

The resolution offered by the committee, that all delegates who should serve and attend for four consecutive years, should be eligible for and should be declared permanent members, was also discussed at length. On motion of Dr. S. O. Vanderpoel, all that portion of the address which related to delegates and permanent membership, was referred to a committee composed of the Chair and two other members, with instructions to report at the next annual meeting. Carried, and the Chair appointed the committee as follows:

Drs. Fisher, of Sing Sing, and Hutchinson and Prout, of Brooklyn.

Dr. Hutchinson. of Brooklyn, called attention to the fact that for two years the name of Drs. Prout and Seeger, of Brooklyn, had been omitted from the published lists of persons eligible for permanent membership, and asked that care should be taken that they are included in the next list, and that they should be marked as eligible since 1872.

The Business Committee reported the following papers by title:

Memoirs of Andrew F. Little and U. G. Bigelow, by Dr. Levi Moore.

Report of Dr. Salvatore Caro, delegate to Pennsylvania State Medical Society.

Report of Robert Newman, delegate to New Jersey State Medical Society.

Resolutions of the Kings County Medical Society.

The Society then took a recess to half-past 3 P. M.

---

*Afternoon Session.*—The Society was called to order at half-past three o'clock, by the Vice-President Dr. H. Jewett.

Dr. William Govan, of the committee appointed to invite the Governor, Lieut.-Governor, and members of the Legislature to take part in the deliberations of the Society, reported that the committee had performed the duty assigned them. The report was accepted and the committee discharged.

Dr. Henry D. Noyes, of New York, read a paper entitled: "Cases of Disease of the Orbit of Unusual Character or Severity."

Among other cases, the doctor mentioned some in which total blindness of one eye had resulted from falls upon and injuries of the frontal prominence. The doctor thought that the blindness in these cases was caused by the force of the blow being transmitted by the orbital plate to the optic foramen, at which point the concussion of the optic nerve was so great as to destroy its ability to transmit luminous impressions.

Dr. Hutchinson, delegate from the Rhode Island State Medical Society, was introduced.

Dr. A. N. Bell read a portion of the report of the Committee on Hygiene, embracing selections from the report of the following counties, viz.: Albany, Cayuga, Chautauqua, Erie, Genesee, St. Lawrance, Montgomery, Ontario, Saratoga, and Kings.

The report was discussed by Drs. Kendall and Govan.

Dr. A. M. Vedder, of Schenectady, gave the history of three cases as follow: "Embolism of the Central Artery of the Retina," "Embolism of Axillary Artery," "Diptheria with Tracheotomy."

Dr. Burge, of Brooklyn, deprecated too early resort to tracheotomy in croup or diptheria. Many cases, he thought, were able to recover without the resort to so hazardous an expedient. He had seen cases recover without the operation, in which death seemed imminent, and, on the other hand, had seen twenty-three operations with but one recovery.

Dr. A. Jacobi protested against allowing a patient to suffer where the operation would give him relief at once. The operation he considered to be by no means dangerous, nor was the presence of a simple hard-rubber trachea tube in the windpipe a source of any serious trouble. If superficial ulcerations of the mucous membrane occurred, they were quite as much a result of the influence of the disease upon the constitution of the tissue as of the presence of a tube. He would as soon think of declining to open the trachea of a child suffering from imperfect oxygenation of its blood as a result of narrowing of its glottis, as he would hesitate to cut down a man hanging by a rope, because it *might* be too late to restore him to life.

Dr. Hutchinson, of Brooklyn, expressed his belief that the operation of tracheotomy was not, *per se*, a dangerous one. It is true that we cannot tell in many cases of croup if death will result, but he was decidedly in favor of giving the patient the benefit of whatever good could come from the procedure.

Dr. F. N. Otis, of New York, next read a paper on "Stricture of the Male Urethra and its Radical Cure."

Dr. Hutchinson, of Brooklyn, remarked that the method of treating strictures advocated by Dr. Otis, if the true one, is one of the most important advances made in surgery in many years.

Dr. Burge, of Brooklyn, wished, at this point, to remark that some years ago he had presented to this Society an instrument for the dilatation of stricture. Subsequent observations had convinced him that it was not worth the snap of his finger.

The Business Committee read by title the report of Dr. Eugene Beach, delegate to the Connecticut Medical Association, which was referred to the Committee on Publication.

*Perityphlitic Abscess.*—Dr. J. W. S. Gouley, of New York, read a paper on "Perityphlitic Abscess due to Perforation of the Appendix Vermiformis, together with Remarks on the Subject of Treatment thereof." The paper was based upon the notes of twenty-four cases, of which the following were not mentioned by either Drs. Lewis, Bull, or Buck, in their papers on this subject, viz.:

Of Dr. Kelsey's, 1,* Dr. Whital, 1,† Dr. J. C. Hutchinson, 2,‡ Dr. H. Bonticou, 2,§ Dr. C. A. Leale, 2,‖ Dr. J. H. Pooley, 1,¶ Dr. J. W. S. Gouley, 1.

Dr. Gouley's case was a man, aged thirty-seven, who had for some time been the subject of hernia, for the relief of which he wore a truss. He frequently suffered from attacks of tenderness, in consequence of which he was often obliged to remove or change his truss. He came under the doctor's care in June of 1873 for treatment during an unusually prolonged and intractable attack of this sort, when his wife reminded him that two years before he had swallowed a fragment of a tooth. He had at that time a swelling in the right illiac fossa, which, after an interval of improvement, became larger, extending over towards median line of abdomen; fever and delirium supervened, and in August it opened spontaneously. No foreign body was noticed in the discharged matter. Improvements which occurred at once resulted in cure in December. In February, 1874, another attack occurred. A consultation was held with Willard Parker, and after a time the tumor was opened freely. A careful examination was made without the discovery of a foreign body. The patient recovered without the recurrence of hernia.

As a result of the study of this, and the history of the other cases reported, Dr. Gouley believed that when a spontaneous discharge of the contents of the abscess occurred, the opening should be enlarged sufficiently to permit a thorough exploration and emptying of its cavity, and that when opened artificially, it should not be done before the seventh day. In a subsequent statement, the doctor called attention to the necessity for the removal, at the same time, of the diseased appendix.

Dr. Thos. F. Rochester, of Buffalo, said that it had been his misfortune to have between fifteen aud twenty cases of this sort in his practice, thirteen of which ended fatally. In these examined the foreign body was found in the majority, either in the

---

* *Medical Record*, December, 1874.

† *Medical Record*, May, 1874.

‡ Communicated.

§ Trans. N. Y. State Med. Society for 1873.

‖ Communicated.

¶ Communicated.

appendix or escaped. The mass was often not a foreign body, properly speaking, but a collection of phosphates derived from the secretions of the intestine, mixed and coated with fæces. He thought it very rare that foreign or other bodies got into a healthy appendix, and thought that there were almost always previous catarrhal symptoms.

Dr. Ernst Krackowizer, of New York, gave his experience in this affection, saying, among other things, that the appendix sometimes becomes fixed by inflammation, when the movements of the intestine are sufficient to produce its dilatation and consequent admission of foreign bodies.

He cited the case of a boy who had fæcal fistula into the bladder and said that the inflammation sufficient to cause the disturbance may have been during the fœtal life of the patient.

Dr. J. V. Kendall, of Baldwinsville, presented ninety-six biliary concretions removed from the gall bladder of a patient who, during life. had no symptoms which indicated the nature of her case.

Dr. Louis Elsberg, of New York, gave the Society a short description of the method and use of "Auscultation of the Œsophagus," and was followed by Dr. Hutchinson, of Brooklyn, who mentioned a case of a child who had swallowed a coin, in which auscultation, by the method spoken of by Dr. Elsberg, indicated the seat of the obstruction.

Dr. Squibb asked what course would be pursued with prize-papers. If the Society reserved the right of publishing them, or allowed the writers, as in the case of other papers, to publish them at their option ?

The President decided that the latter course was consistent with the by-laws.

Dr. E. M. Moore, of Rochester, exhibited to the Society the extremeties of two ulnæ from cases of fracture of the wrist—one was removed at the time of the injury, and was the specimen shown by him at a meeting of the Medical Society of the County of New York, in 1873. The other was removed a short time since from a patient who had about six or seven years before had the peculiar fracture, which Dr. Moore has before called attention to. Both cases showed the nature of the injury as regards the separation of the triangular ligament for the pit of the styloid process, bringing away a scale of bone and converting the process into a sharp point, which punctures the annular ligament or has the latter bent sharply over its apex.

The Society then adjourned to meet in the Assembly Chamber at eight o'clock, to listen to the annual address of the President.

---

*Evening Session..*—The Society met in the Assembly Chamber at eight o'clock, to listen, agreeably to custom, to the President's annual address.

Referring to the various subjects which had been considered by his predecessors, Dr. Fisher chose for his paper the history of the Society since the first steps were taken towards its organization in 1796 in Saratoga County.

At the close of the address, the members and guests, to the number of three hundred or more, adjourned to the house of Governor Tilden, and after presentation to the Governor and his family, partook of an elegant supper.

---

*Third Day.—Morning Session.*—The Society being called to order at half-pest nine o'clock, the meeting was opened with prayer by the Rev. Dr. Bridgman.

The President announced that the committee to wait on the Legislature in reference to the subject of change of time of meeting would be Dr. Quakenbush.

Dr. Squibb understood that the Society had an organic right to change its time of meeting without reference to the Legislature.

The President said the Society had to give notice of its intention.

The Committee on Arrangements and Receptions reported a list of delegates and invited guests present.

The Committee on Ethics, through Dr. T. Hun, reported that they had entertained the complaint of J. C. C. Dove, of Dutchess County, and for want of other than *ex parte* evidence, declined to act. It appeared to them that the County Society in question had not been duly notified.

Dr. E. H. Parker, of Poughkeepsie, said that he was aware that the County Society *had* been duly notified in the manner required, and urged that some action should be taken at the earliest possible moment, since, until facts to the contrary were known, the petitioner might be the aggrieved person.

Dr. Eliot moved that the report be referred back to the committee with power.

Dr. Parker was quite willing that this course should be pursued, but objected to the "with power." The case, he thought, was an important one, and the decision might establish a precedent; therefore, he thought the decision of the question should rest with the Society. Dr. Eliot accepted Dr. Parker's amendment, and the motion was carried.

Dr. Kendal, Chairman of Committee to invite the Health Committee of the Legislature, reported that they had discharged that duty, and on motion their report was accepted.

The Committee appointed to audit the Treasurer's account, reported that it had been found correct as per vouchers presented.

On motion of Dr. Eliot, the report of the committee was accepted.

The Committee on Nominations reported the list of officers, delegates. etc., for the ensuing year, of which the following are the most important items:

*For President*—Dr. Thomas F. Rochester, of Buffalo; *Vice-President*, Dr. Ellsworth Eliot, of New York; *Secretary*, Dr. E. R. Hun, of Albany; *Treasurer*, Dr. Charles F. Porter, of Albany.

*Censors.*—The list of censors remains about the same as last year, with the exception that J. C. Hutchinson, of Brooklyn, is appointed in place of E. R. Squibb, in the southern district; and C. C. Wyckoff, of Buffalo, in place of L. B. Cotes, of Batavia.

*Committee on Correspondence*—The same as last year.

*Committee on Statistics*—The same as last year.

*Committee on Prize Essays*—Henry W. Dean, of Rochester; Julius F. Miner, of Buffalo; William S. Ely, of Rochester.

*Committee on Publication*—A. E. M. Purdy and Ellsworth Eliot, of New York, and E. R. Hun, of Albany.

*Censor of Syracuse Univ., Medical Department*, same as last year.

The committee also presented the following Preamble and Resolution, which were unanimously adopted:

*Whereas*, After a continuous period of service as Secretary of this Society for ten years, Dr. W. H. Bailey feels constrained to decline the further acceptance of the office, it is, therefore,

*Resolved*, That the acknowledgments of the Medical Society of the State of New York are hereby conveyed to Doctor William H. Bailey for the self-sacrificing and eminently successful manner in which, by his courtesy, skill and ability he has discharged the arduous duties incident to his position; and that this Resolution be published in the proceedings of this Society.

*Resolved*, For the purpose of complying with a by-law of the American Medical Association, that the delegate from this Society whose name heads the list, be regarded as the member of the Nominating Committee from the State of New York in that body; and in the event of his absence, that the delegate in attendance whose name is next in order shall be chosen to that position.

On motion of Dr. Eliot, the Report was accepted, and the Resolutions adopted.

Following the election, the Committee on Prize Essays declared the following subjects to be competed for during the coming year:

For the Merrit Cash Prize—"Transfusion, Historically, Experimentally, and Critically Considered."

For the Brinsmade Prize—the subject optional with the candidate.

The President announced that the Committee on Hygiene would remain the same as last year. He also mentioned the death, in August last, of Samuel Shumway, M. D., a member of the Society.

On motion of Dr. Eliot, the Society directed the retiring President to fill all vacancies that might occur in the list of delegates.

Dr. Squibb offered a series of resolutions relative to the publication of the Transactions which, after some discussion, were adopted.

Dr. Frank H. Hamilton, of New York, gave his experience in the use of hot water by immersion in the treatment of amputations and other surgical affections in the Reception Hospitals and St. Francis Hospital, in New York, during the past three years. (See former No. of the *Medical Record.*)

Dr. Squibb said that it was certainly gratifying to his complacency to have the views expressed many years ago in an article contributed to the *Amer. Journal of Med. Sciences* receive such endorsement. He offered also a suggestion which came first from French surgeons, that a small quantity of aromatic wine should be added to the water, not only because of its odorus nature, but because its ingredients possessed disinfectant properties.

Dr. Krackowizer, of New York, had had some experience in the German Hospital in the employment of this measure and considered the use of hot water mostly restricted to the primary stage of injuries. After cicatrization had commenced, he thought the œdema produced by the heat, moisture, and dependent posture combined tended to interfere with recovery. Dr. Hamilton had spoken of the restriction of the application to the parts below the upper arm and thigh, but he knew no reason why immersion of the whole body might not overcome the difficulty when other parts were the seat of trouble. Hebra had done this in the case of extensive burns, and the patients had been kept under water for weeks together.

Dr. Quackenbush reported that the committee appointed to wait on the Legislature had presented the subject of change of time of meeting to Hon. Mr. Husted, who had promised to see that it received early attention. The Business Committee read the following papers by title, and they were referred to the Committee on Publications:

"Case of Puerperal Convulsions, Apoplexy," by Dr. Cartwright.

"Case of Fracture of Humerus."

"Case of Gall Stones."

"Removal of Breast by Knife, and afterwards by Elastic Ligature," by Dr. Abbot.

Dr. Sherman, of Ogdensburg, narrated cases of "Fracture and Dislocation of Accrominal Extremity of Clavicle."

Dr. E. M. Moore declared his interest in these cases which are truly rare. He had no knowledge of the results of this method in dislocation until two years ago. Last summer he received a letter from Dr. Prince, of Illinois, reporting the successful issue. Two months later he had another case which resulted so favorably that it was difficult to tell which was the injured shoulder. These four case were the only ones of which he had heard.

Dr. Hamilton thought the gentleman had not made a distinction between two varieties of this injury, one complete, where the outer extremity of the calvicle over-rides the accromion, the other, where the luxation was incomplete. Of the two, the former is, of course, the most serious, and the perfect cure questionable.

Dr. Rochester had treated successfully a case of complete dislocation of sternal end, when the attention was attracted by the interference with breathing caused by pressure of the extremity of the calvicle against the trachea.

Dr. Sherman said that in both the cases reported by him the dislocation was complete.

Dr. Squibb moved that Professors Hamilton and Dalton be requested to furnish the substance of their remarks to the Committee for publication in the Transactions. Carried.

MISCELLANEOUS.

The Committee on Credentials, through Dr. Wey, reported that two hundred and fifty-nine names were registered.

There being no further business, the minutes of the morning's session were read and approved, and the Society adjourned *sine die.*—*Medical Record.*

